# X-ray is not inferior to CT in terms of F1 score in the diagnosis of foreign body aspiration: a recall, precision and F1 score performance analysis based on bronchoscopically proven cases

**DOI:** 10.1016/j.jped.2025.101430

**Published:** 2025-08-22

**Authors:** Fatma Sarac, Mehmet Yazici

**Affiliations:** Department of Pediatric Surgery, Basaksehir Cam and Sakura city Hospital, Istanbul, Turkey

**Keywords:** Aspiration, Airway, Bronchoscopy, Child, Foreign body

## Abstract

**Objective:**

In this study, we aimed to evaluate the diagnostic accuracy of X-ray and CT by using the F1 score with its non-inferiority margin in patients who underwent bronchoscopy with suspected diagnoses of foreign body aspiration (FBA).

**Methods:**

All children aged under 18 who underwent bronchoscopy with suspected diagnoses of FBA between June 2020 and December 2023 were included. The patient’s medical records were examined retrospectively.

**Results:**

A total of 310 patients were included. Foreign bodies were most frequently located in the right main bronchus (47.52 %). The bronchoscopy (-) rate was 18 % in patients who had X-ray (+), 24 % in those who had CT (+), and 15 % in those who had X-ray (+) and CT (+). Chest x-rays were found to exhibit 42 % sensitivity, 74 % specificity, a positive predictive value (PPV) of 61 %, a negative predictive value (NPV) of 51 %, accuracy of 66 %, precision: 0.63, recall: 0.82, and F1 score: 0.71. Analysis showed that CT exhibited 82 % sensitivity, 37 % specificity, a PPV of 75 %, NPV of 47 %, an accuracy rate of 69 %, precision: 0.83, recall: 0.76, and F1 score: 0.79. In the present study, the diagnostic F1 score was calculated as 0.79 for CT and 0.71 for X-ray.

**Conclusion:**

Despite a negative bronchoscopy rate of 34.83 % in this study, since the authors observed no severe complications or mortality, the authors recommend that it be performed on all patients with suspected foreign body aspiration. When a 10 % non-inferiority margin was applied, X-ray was found to be not inferior to CT in terms of F1 score.

## Introduction

Foreign body aspiration (FBA) is an important preventable condition and cause of death in children [1]. It is also a serious societal problem as a domestic incident seen particularly in children aged 1–3 and requiring emergency diagnosis and treatment. Factors implicated in FBA include the fact that children of this age tend to use their mouths to explore their surroundings, that the neuromuscular mechanism that enables them to chew food properly and protect the airway is not yet fully matured, and insufficient supervision by adults. Other likely factors in FBA may include dental development and poor parental/societal understanding and awareness of the serious risks certain foods may entail if either given to or in reach of children. It is more common in boys than girls [1, 2, 3, 4, 5, 6, 7].

Foreign bodies aspirated are thought to vary depending on countries’ feeding habits, traditions, and sociocultural and religious characteristics [2,8,9]. The most frequently aspirated organic objects include foods such as peanuts, seeds, hazelnuts, sunflower seeds, and carrots, while inorganic objects include small toys, pen caps, pieces of plastic, and pins/needles [2,8, 9, 10, 11, 12].

Due to the anatomical and physiological characteristics of the airways, most foreign bodies are located in the right main bronchus [6,7,13,14]. The type of material aspirated, its location, the level of obstruction, the patient’s age, and the time of diagnosis all affect the severity of the clinical manifestation and physical examination findings [2, 3, 4, 5,10]. Removal of the foreign body from the airways with rapid and correct diagnosis is highly important. Rigid bronchoscopy represents the gold standard in the treatment of FBA. The purpose of this study is to describe the management of FBA in our clinic, our region’s most important bronchoscopy center since it first entered into operation.

## Materials and methods

Children aged under 18 who underwent bronchoscopy with suspected diagnoses of FBA between June 2020 and December 2023 at the pediatric surgery clinic were included in the study. Approval was obtained from the ethics committee of the Hospital on 27.12.2023. The procedures used in this study adhere to the tenets of the Declaration of Helsinki (Protocol number 2023–668).

All patients who underwent bronchoscopy were scanned via the hospital data system and clinical surgery records and were evaluated in terms of age, sex, aspiration history and material, presentation symptoms, physical examination findings, imaging tests, radiological findings, bronchoscopy results, and post-bronchoscopy lengths of hospital stay. Based on the X-ray and CT data, the authors calculated sensitivity, specificity, recall, precision, accuracy, and F1 scores.

### How were X-ray and CT evaluated?

All chest X-rays and thoracic CT scans were independently reviewed before pediatric surgery consultation by a senior radiologist who was blinded to the patients’ bronchoscopy results and clinical outcomes. X-ray (+) was defined as the presence of radiographic findings suggestive of foreign body aspiration, including unilateral hyperinflation, mediastinal shift, air trapping, or localized atelectasis. X-ray (-) referred to radiographs showing no such abnormalities. Similarly, CT (+) was defined as the direct visualization of a foreign body or indirect findings consistent with obstruction (e.g., air trapping, collapse, or hyperinflation), whereas CT (-) referred to scans without such indicators. All imaging assessments were performed prior to consultation with pediatric surgery to eliminate interpretive bias. The decision to proceed with bronchoscopy was based on a combination of clinical presentation (e.g., witnessed choking, persistent symptoms, abnormal auscultation) and radiological findings. A chest X-ray was routinely performed on all patients. Thoracic CT, however, was reserved for selected cases where it was deemed necessary—particularly in patients with delayed presentation, those requiring differential diagnosis, or cases with a strong clinical suspicion of FBA despite normal X-ray findings.

### Calculation of scores

Precision is calculated by dividing the number of true positives (TP) by the sum of true positives and false positives (TP + FP), indicating the proportion of correctly predicted positive cases out of all positive predictions. Recall, on the other hand, is determined by dividing true positives (TP) by the sum of true positives and false negatives (TP + FN), representing the ability to identify all actual positive cases. The F1 score harmonizes precision and recall. A 10 % non-inferiority margin was selected based on commonly accepted thresholds in diagnostic accuracy studies, particularly in pediatric populations where balancing radiation exposure and diagnostic value is crucial. Previous studies evaluating non-inferiority in diagnostic tests have used similar margins to reflect clinically acceptable differences without compromising patient safety. This threshold was deemed reasonable for comparing two modalities with differing risk-benefit profiles.

### Statistical analysis

Descriptive statistics were presented as mean, standard deviation, minimum and maximum values for continuous data. The normality of distribution for continuous data in statistical comparisons was assessed using the Kolmogorov-Smirnov test, the *t*-test being applied for parametric data and the Mann-Whitney U test for non-parametric data. The Kruskal-Wallis test was applied in group comparisons, and the Mann-Whitney U test for two-way comparisons. Spearman correlation analysis was applied for the examination of data co-variation. P values lower than 0.05 were regarded as significant. SPSS version 21.0 software (SPSS Inc., Chicago, IL, USA) was used for statistical analyses. The diagnostic value of X-ray and CT was assessed using recall, precision, and F1 score to determine non-inferiority.

## Results

Three hundred ten patients, 212 (68.4 %) boys and 98 (31.6 %) girls, examined between June 2020 and December 2023 were included in the study. Thirty-seven patients (11.93 %) were younger than one year, 220 (70.96 %) were aged 1–2, 22 (7.1 %) were aged 2–5, and 31 (10 %) were older than five (Table 1). The complaints and auscultation findings are given in Table 2.

**Table 1:** The patients’ ages.

Age	n	%
< 1 (0–11 months)	37	11.93
1–2 years	220	70.96
2–5 years	22	7.1
> 5 years	31	10
**Total**	**310**	**100**

**Table 2:** Patients' complaints and physical examination findings.

Complaints	Number of patients (n)	Percentage ( %)
Choking	135	43.5
Cough	109	35.16
Wheeze	29	9.35
Dyspnea	22	7.1
Other	15	4.8
Total	310	100
**Chest Auscultation**		
Decreased respiratory sounds	137	44.2
Normal	91	29.35
No information	82	26,45
Total	310	100

Chest x-ray was performed on all patients (*n* = 310). A difference in inflation was present in 155 patients at lung radiography, but not in the other 155. Foreign bodies were observed during bronchoscopy in 127 of the patients with increased ventilation at chest x-ray, but not in the remaining 28. Foreign bodies were present at bronchoscopy in 75 patients with no difference in ventilation at chest x-ray, but not in the other 80. Chest x-rays were found to exhibit 42 % sensitivity, 74 % specificity, a positive predictive value (PPV) of 61 %, a negative predictive value (NPV) of 51 %, accuracy of 66 %, precision: 0.63, recall: 0.82, and F1 score: 0.71 (Table 3).

**Table 3:** Comparison of chest radiography and bronchoscopy.

Chest x-ray	Bronchoscopy FB (+)	Bronchoscopy FB (-)	Total
FB (+)	127	28	155
FB (-)	75	80	155
Total	202	108	310

FB (+): difference in inflation on chest x-ray; FB (-): No difference in inflation.

Sensitivity: 42 %; Specificity: 74 %; PPV: 61 %; NPV: 51 %; Accuracy rate: 66 %, Precision: 0.63; Recall: 0.82; F1 score: 0.71.

Thoracic computed tomography (CT) was performed on 91 (29.35 %) of the 310 patients. Foreign bodies were present in 70 patients at CT, and not in 21. Foreign bodies were not detected at bronchoscopy in 17 of the patients in whom such bodies were reported at In addition, foreign bodies were detected at bronchoscopy in 11 of the patients with no such bodies reported at CT. Analysis showed that CT exhibited 82 % sensitivity, 37 % specificity, a PPV of 75 %, NPV of 47 %, an accuracy rate of 69 %, precision: 0.83, recall: 0.76, and F1 score: 0.79 (Table 4). In the present study, the diagnostic F1 score was calculated as 0.79 for CT and 0.71 for X-ray. When a 10 % non-inferiority margin was applied, X-ray was found to be not inferior to CT in terms of F1 score.

**Table 4:** Comparison of thoracic computed tomography and bronchoscopy.

Thorax CT	Bronchoscopy		
	FB (+)	FB (-)	Total
FB (+)	53	17	70
FB (-)	11	10	21
Total	64	27	91

FB, Foreign body; Sensitivity: 82 %; Specificity: 37 %; PPV: 75 %: 47 %; Accuracy rate: 69 %; Precision: 0.83; Recall: 0.76; F1 score: 0.79.

The bronchoscopy (-) rate was 18 % in patients who had X-ray (+), 24 % in those who had CT (+), and 15 % in those who had X-ray (+) and CT (+) (Table 5).

**Table 5:** Bronchoscopically proven negative rates (Negative rates demonstrated by bronchoscopy).

	Bronchoscopy (-)	Number of patients	Bronchoscopy (-) rate
X-ray (+)	28	155	18 %
CT (+)	17	70	24 %
X-ray (+) and CT (+)	8	54	15 %

Foreign bodies were located in the right main bronchus in 96 patients (47.52 %) during bronchoscopy, in the left main bronchus in 73 (36.13 %), in the laryngotracheal region in 17 (18.41 %), in the bilateral main bronchi in 11 (5.44 %), in the trachea + one of the main bronchi in four (1.98 %), and in the laryngotracheal region + both main bronchi in one. No foreign body was detected at bronchoscopy in 108 patients (34.83 %) (Table 6).

**Table 6:** Foreign body localizations.

FB location	n	%
Right main bronchus	96	47.52
Left main bronchus	73	36.13
Larynx/trachea	17	8.41
Left + right main bronchi	11	5.44
Trachea + one main bronchus	4	1.98
Larynx/trachea + left + right bronchi	1	0.49
No FBA	108	34.83
**Total**	**310**	**100.00**

The aspirated material was organic in 169 cases (83.66 %). These consisted of peanuts in 55 cases (32.54 %), hazelnuts in 41 (24.26 %), sunflower or pumpkin seeds in 18 (10.65 %), while 15 (8.87 %) involved walnut, and the others almond, apple, carrot, maize, chickpea, and chestnut. Inorganic matter aspiration was present in 31 cases (15.34 %). These consisted of items such as pins (4), pieces of glass (4), pen caps (4), drawing pins (3), earrings, pieces of plastic, beads, screws, and Lego bricks. No foreign body was detected in 108 patients (34.83 %). No aspiration material information was available for the two patients. A statistically significant difference was present between organic and inorganic FBA (*p* < 0.001). There was no significant relationship between age or sex and the type of material aspirated (*p* = 0.124 and *p* = 0.366, respectively).

Treatable complications developed in 24 patients (7.7 %) who underwent bronchoscopy, including bronchospasm in 16 (5.16 %) bronchial bleeding in five, hypoxia-low saturation in two, and pneumo-mediastinum in one. *Re*-bronchoscopy was required in 17 cases (5.48 %) since the procedure could not be completed originally, due to the inability to withdraw the foreign body completely in 10 patients, since the symptoms persisted in four, and due to bronchospasm, bronchial bleeding, and hypoxia in one patient each. Twenty patients required follow-up in intensive care, two of whom were intubated, following the bronchoscopy procedure.

The minimum time elapsing to presentation to the hospital following the onset of FBA was 1 hour, with a maximum value of 3 years, and a median value of 24 h. The length of post-bronchoscopy hospital stay values were at a minimum of 1 hour, median of 24 h, and maximum of 2 months**.** A significant weak correlation was determined between time to presentation and length of hospital stay (*p* = 0.001). The length of the stay exhibited a mild increase as the time for presentation increased.

A significant correlation was observed between the length of stay post-bronchoscopy and the anatomical location of the foreign body (*p* = 0.017). At two-way group comparisons, statistically significant differences were observed between patients with no foreign bodies and those with foreign bodies in both bronchi (*p* = 0.018), those with foreign bodies in the trachea/larynx and those with bodies in both bronchi (*p* = 0.002), those with foreign bodies in the right main bronchi and those with foreign bodies in the left main bronchi (*p* = 0.028), and those with foreign bodies in the right main bronchi and those with foreign bodies in the bilateral bronchi (*p* = 0.012) (*p* < 0.05).

## Discussion

This study evaluates the diagnostic performance of X-ray and CT in foreign body aspiration (FBA) cases confirmed via bronchoscopy. A total of 310 patients were included, with foreign bodies most frequently found in the right main bronchus. Organic materials like peanuts were the most aspirated objects. The F1 score for CT was 0.79, while X-ray had a score of 0.71. Applying a 10 % non-inferiority margin, X-ray was determined to be not inferior to CT, highlighting its potential as a diagnostic tool.

Foreign body aspirations are causes of domestic accidents frequently seen in children. Patients typically present with histories of sudden onset cough and respiratory distress while eating or playing with toys [2]. The great majority of FBAs occur in children under three, the highest incidence being between the ages of one and two years [2,4,6,7,9,10,11,15]. The higher incidence among boys is attributed to their more adventurous natures [1, 2, 3, 4, 5, 6, 7]. In agreement with the previous literature, approximately 82 % of these patients were aged two or younger, and two-thirds (68.4 %) of cases involved boys.

The presenting symptoms of FBA are most commonly due to impaired or obstructing respiration. The most common reasons for presentation are symptoms such as sudden-onset cough, grunting, and wheezing, the aspiration event being witnessed, and choking [1,2,7,9, 10, 11, 12]. The most common reason for presentation in the present study, seen in 43.5 % (*n* = 135) of patients, was aspiration being witnessed by the family and accompanying choking. This was followed by a sudden-onset cough at 35.16 % (*n* = 109). On physical examination, 52 % of patients with foreign bodies in the right main bronchus and 50 % of patients with foreign bodies in the left main bronchus on bronchoscopy had decreased breath sounds on auscultation. It’s important to note that in the present study all auscultation was performed by a senior pediatric surgeon.

Complementary examinations such as chest X-rays, thoracic CT, and flexible bronchoscopy are performed in cases of suspected FBA to reduce negative bronchoscopy rates due to its severe potential risks. The chest x-ray is the first step test in diagnosis. However, chest X-rays are not specific in the diagnosis of FBA, and a normal chest X-ray does not always exclude a diagnosis of FBA in patients with a history of such aspiration and positive physical examinations. Sattar et al. reported 66.6 % sensitivity, 50 % specificity, 89.6 % PPV, 18.7 % NPV, and 64.4 % accuracy for chest X-rays [16]. Chest X-rays were taken of all the patients and exhibited 42 % sensitivity, 74 % specificity, 61 % PPV, 51 % NPV, and 66 % accuracy.

Thoracic CT was performed in 91 patients (29.35 %) in the present study, predominantly in cases where there was a discrepancy between the clinical history or physical examination findings and inconclusive or normal chest X-rays. Specifically, CT was requested when there was a strong clinical suspicion of FBA (e.g., witnessed choking, persistent cough, unilateral decreased breath sounds), but without definitive radiographic evidence. This selective and non-standardized use of CT, often in a delayed or referral context, may have contributed to the relatively low specificity(37 %) and higher false-positive rate (24 %) observed in the present results. These findings differ markedly from those reported by El Khoury et al. [17] (sensitivity: 99 %, specificity: 92 %) and Nasir and Subha [7] (sensitivity: 100 %, specificity: 66.7 %, NPV: 100 %) where CT was likely performed earlier in the diagnostic process and under more controlled conditions. Based on these findings, the authors propose that thoracic CT may be better suited as a tool for differential diagnosis in atypical or prolonged cases rather than as a routine modality, especially given the comparable diagnostic value of X-ray as demonstrated by the non-inferior F1 score (0.71 vs. 0.79). This interpretation is intended to support clinical decision-making and reduce unnecessary radiation exposure and overuse of CT, particularly in pediatric populations.

Due to the anatomical and physiological characteristics of the airways, the majority of foreign bodies are detected in the right main bronchial tree [7,8,10,11]. Consistent with the previous literature, foreign bodies were detected in the right main bronchi in 47.52 % of cases in this study, and in the left main bronchi in 36.13 %, the difference being statistically significant (*p* = 0.028).

A significant difference was determined between organic and inorganic FBAs (*p* < 0.001), consistent with the previous literature 2, 4, 7, 9, 10, 12, 18, 19. No significant associations were observed between age or sex and the type of material aspirated (*p* = 0.124 and *p* = 0.366, respectively).

The standard technique for removing bronchial foreign bodies in children is rigid bronchoscopy, used for both diagnosis and treatment and performed under general anesthesia. Its success rate exceeds 97 %. However, severe complications such as laryngeal edema, tracheal and bronchial lacerations, and even cardiac and respiratory arrest may occur. The rate reported in the literature ranges between 2 % and 22 %. Negative bronchoscopy rates between 10 % and 61 % have also been reported in the literature [6]. Ayed et al. reported negative bronchoscopy rates of 12.3 % and Güler et al. one of 38.2 % [13,18].

No mortality occurred in the present study. The complication rate was 7.7 % (*n* = 24) and the re-bronchoscopy rate was 5.48 % (*n* = 17). The negative bronchoscopy rate in this research was 34.83 % (*n* = 108). These complications, largely consisting of bronchospasm, bronchial bleeding, and hypoxia, were mild and treatable. These patients underwent short-term follow-up in the pediatric intensive care unit for purposes of close observation. Only two patients were intubated and underwent longer-term observation. However, respiratory distress was present in half of these patients pre-bronchoscopy. It was therefore unclear whether the complication occurred from the aspiration or from bronchoscopy during surgery. Nonetheless, the total complication rate was subjected to analysis. The authors attribute the high negative bronchoscopy rate (34.83 %) to the majority of patients being referred to this center for bronchoscopy from other institutions since ours is the largest tertiary hospital in the region and a main center in which bronchoscopy can be performed.

In conclusion, FBA is an important condition capable of causing morbidity and mortality in children. Suspicion is the most important step in the diagnosis of FBA, with its widespread and non-specific symptoms associated with the respiratory system, particularly in children aged 1–3. The aspiration being witnessed, decreased unilateral respiratory sounds on the involved side of involvement, and unilateral hyper-aeration being observed on x-rays all represent powerful evidence of FBA. However, the only means of definitely diagnosing FBA in children is rigid bronchoscopy. This is quite a safe procedure in suitably qualified hands.

Despite a negative bronchoscopy rate of 34.83 % in this study, since the authors observed no severe complications or mortality, we recommend that it be performed on all patients with suspected foreign body aspiration. F1 score indicated that X-ray was not inferior to CT. Radiological images exhibiting a normal appearance do not necessarily mean that no foreign body is present. Therefore, bronchoscopy should be performed in radiologically negative patients who have persistent clinical suspicion, such as ongoing respiratory symptoms, witnessed aspiration, or abnormal physical examination findings. It is of course particularly important that the bronchoscopy procedure be performed by qualified individuals and that intensive care requirements should also be considered. If complication management is not possible, then the patient should be transferred to a suitable clinic. Furthermore, the potential risks associated with imaging modalities in pediatric patients warrant consideration. CT scans involve higher radiation exposure, which is particularly concerning in children. Additionally, transporting clinically unstable patients to the radiology unit may delay diagnosis and treatment. Transfers from external centers can further prolong intervention times, potentially increasing the risk of complications.

## Limitations

There are several limitations that should be addressed. First, due to its retrospective design, the study potentially introduces selection bias. Intergroup comparisons of patients with foreign bodies in other parts of the airways than the right and left main bronchi were not highly significant due to the low numbers of such cases. Comparisons with larger patient numbers would yield more accurate results. Since ours is a top-level referral hospital, the majority of patients were referred from external hospitals for direct bronchoscopy, their tests having already been performed. The authors did not therefore perform test planning in the majority of cases. Additionally, the selective use of CT in referred cases and lack of standardized imaging protocols may have introduced bias, potentially limiting the generalizability of the conclusion regarding the diagnostic role of CT versus X-ray in suspected FBA.

## Funding

The authors did not receive support from any organization for the submitted work.

## Ethics approval

Approval was obtained from the ethics committee of Başakşehir Çam and Sakura City Hospital on 27.12.2023. The procedures used in this study adhere to the tenets of the Declaration of Helsinki (Protocol number 2023–668).

## Informed consent

Due to the retrospective nature of the study, Başakşehir Çam and Sakura City Hospital Local Ethical Committee (project number: 2023–668**)** waived the need to obtain informed consent.

## Data and/or code availability

The present data is in the archives of “Başakşehir Çam and Sakura City Hospital’’. Datasets used and/or analyzed during the current study are available from the corresponding author upon reasonable request.

## Authors’ contributions

Conceptualization: FS, MY. Data curation: FS, MY. Formal analysis: FS, MY. Methodology: FS, MY. Project administration: FS. Visualization: FS. Writing original draft: FS. Writing review and editing: FS.

## Use of AI and AI-assisted technologies statement

Generative AI and AI-assisted technologies were not employed during the writing of this paper.

## Conflicts of interest

The authors declare no conflicts of interest.
